# Effectiveness of Meningococcal B Vaccine against Endemic Hypervirulent *Neisseria* *meningitidis* W Strain, England

**DOI:** 10.3201/eid2202.150369

**Published:** 2016-02

**Authors:** Shamez N. Ladhani, Marzia Monica Giuliani, Alessia Biolchi, Mariagrazia Pizza, Kazim Beebeejaun, Jay Lucidarme, Jamie Findlow, Mary E. Ramsay, Ray Borrow

**Affiliations:** St. George’s University of London Paediatric Infectious Diseases Research Group, London, UK (S.N. Ladhani);; Public Health England, London (S.N. Ladhani, K. Beebeejaun, M.E. Ramsay);; GSK Vaccines, Siena, Italy (M.M. Giuliani, A. Biolchi, M. Pizza);; Public Health England, Manchester, UK (J. Lucidarme, J. Findlow, R. Borrow)

**Keywords:** *Neisseria meningitidis*, meningococcal W disease, ST11 clonal complex, prevention, meningococci, serum bactericidal antibody activity, immunization, endemic, meningitis, Bexsero, Menveo, England, vaccine, bacteria

## Abstract

Serum samples from children immunized with a meningococcal serogroup B vaccine demonstrated potent serum bactericidal antibody activity against the hypervirulent *Neisseria* *meningitidis* serogroup W strain circulating in England. The recent introduction of this vaccine into the United Kingdom national immunization program should also help protect infants against this endemic strain.

Invasive meningococcal disease (IMD) has been declining in the United Kingdom since the early 2000s (*1*). Historically, serogroup W *Neisseria* *meningitides* (MenW) have been causal organisms for 1%–2% of IMD cases annually. An increase in invasive MenW disease associated with travel to the Hajj pilgrimage route during 2000–2002 was rapidly controlled after the introduction of mandatory vaccination for pilgrims (*2*). Since 2009, however, laboratory-confirmed MenW cases in England have increased each year across all age groups after rapid spread of a single endemic hypervirulent sequence type (ST) 11 clonal complex (MenW:cc11) strain (*3*). This strain has caused severe illness with unusual clinical manifestations and, for the first time in more than a decade, was associated with fatal outcomes among infants and young children.

Since the Hajj outbreak, several countries in Latin America, Africa, and the Far East have reported an increase in MenW disease and ongoing endemic transmission (*3*). In Chile, MenW has replaced serogroup B (MenB) as the most prevalent cause of IMD, identified in 58% of cases in 2012 (*4*). In Europe, an increase in MenW disease has not been observed in other countries although, in 2012, a cluster of cases related to MenW:cc11 in France was associated with travel to sub-Saharan Africa (*5*).

In England, the meningococcal quadrivalent conjugate vaccine (MenACWY, covering serogroups A, C, W, and Y) has historically been recommended for high-risk persons and travelers to disease-endemic regions and for controlling outbreaks (*6*). Beginning on September 1, 2015, a novel, protein-based, multicomponent vaccine, Bexsero (GSK Vaccines, Siena, Italy), has been offered as part of the routine immunization program in the United Kingdom; the vaccine is given to infants in 3 doses at 2, 4, and 12 months of age (https://www.gov.uk/government/publications/menb-vaccination-introduction-from-1-september-2015). Bexsero is composed of NHBA (neisserial heparin binding antigen), NadA (*Neisseria* adhesin A) and fHbp (factor H binding protein), with meningococcal outer membrane vesicles from MenB strain from an outbreak in New Zealand (*7*). Immunization with Bexsero induces bactericidal antibodies against all vaccine antigens (*8*). Although this vaccine has been licensed for prevention of MenB disease (the most prevalent capsular group causing IMD in Europe), alleles for some or all of the vaccine antigens are also found among non-MenB meningococci, independently of the capsule. Therefore, antibodies raised by these antigens could induce complement-mediated killing of other meningococcal groups, including the endemic MenW cc11 strain. 

Recently, we reported the predominance of non–cross-protective PorA (P1.5,2) and fHbp variants (variant 2 peptide 22) among endemic MenW:cc11 isolates (*3*). The other primary Bexsero antigens (NadA and NHBA), however, could potentially afford protection. We therefore assessed 1) the NadA and NHBA genotypic status of endemic MenW:cc11 isolates, and 2) the serum bactericidal antibody (SBA) activity against clinical MenW:cc11 isolates using serum samples from infants immunized with Bexsero.

## The Study

A total of 73 invasive MenW:cc11 isolates from England and Wales were received by the Public Health England Meningococcal Reference Unit during July 2010–June 2013. These isolates were queried within the Meningitis Research Foundation Meningococcus Genome Library (http://pubmlst.org/perl/bigsdb/bigsdb.pl?db = pubmlst_neisseria_mrfgenomes) for *nadA* and *nhba* and, where present, their respective allelic and peptide variants.

We used an SBA using human complement (hSBA) against 6 invasive MenW:cc11 isolates from patients 4 months–91 years of age in whom meningitis or septicemia was diagnosed in different regions of England and Wales during 2011–2012. SBA titers were expressed as the reciprocal of the serum dilution corresponding to >50% bacterial killing. We used pooled serum samples from phase 2 clinical trials involving infants immunized with Bexsero at 2, 3, and 4 months or 2, 4, and 6 months and after administration of a booster at 12, 18, or 24 months (*8*). Pooled prevaccination serum samples from 180 infants were used as negative controls, and pooled serum samples from 10 randomly selected adolescents who received a single MenACWY conjugate vaccine (GSK Vaccines) dose as positive controls.

Of the 6 isolates tested, 4 possessed *nadA* allele 5 (for peptide NadA-2/3.6) and *nhba* allele 17 (for NHBA peptide 29). Of the remaining 2 isolates, 1 isolate had a *nadA* allele (allele 146) that differed at a single nucleotide (C476A), causing a single amino acid change (T159K; peptide NadA-2/3.130); the other isolate had an *nhba* allele (allele 72) that differed at a single nucleotide (A376C), causing a single amino acid change (T126P; peptide 96).

hSBA titers were high and were >1:32 against all 6 MenW isolates, independently of the immunization schedule (Table). After the booster, higher hSBA titers were obtained than those from primary immunization, and similar responses were comparable to those among adolescents who had received a single MenACWY dose. Preimmunization serum samples showed no detectable hSBA titers against any of the 6 isolates (Table).

**Table:** Bactericidal antibody titers in pooled serum samples from infants vaccinated with Bexsero and adolescents immunized with Menveo against 6 invasive clinical *Neisseria meningitidis* serogroup W isolates in England and Wales, UK, during 2011–2012*

Isolate	Adolescents receiving Menveo			Infants receiving Bexsero				
	Positive control†			Negative control‡	Pool 1§	Pool 2¶	Pool 3#	Pool 4**
	Before	After						
M11–240417	<16	256		<2	64	128	>128	>128
M11–240427	<16	128		<2	32	32	64	64
M11–240802	<16	512		<2	32	>64	>64	>64
M12–240016	<16	256		<2	32	32	64	128
M11–240798	<16	512		<2	>64	>64	>64	>64
M12–240754	<16	256		<2	64	64	>64	>64

*Human serum bactericidal antibody titers against 6 meningococcal group W clinical isolates belonging to ST-11 clonal complex using pooled serum from adolescents immunized with the MenACWY conjugate vaccine, Menveo, and from infants immunized with the protein-based, multicomponent meningococcal vaccine, Bexsero. †Pooled serum samples from adolescents 11–17 years of age before and 1 month after receiving a single dose of MenACWY (Menveo) conjugate vaccine (n = 10). ‡Pooled prevaccination serum samples from infants at 2 months of age (n = 180) (control). §Pooled serum samples from infants who received 3 doses of vaccine at 2, 3, and 4 months of age and had a blood sample taken 1 month after the last dose of vaccine (n = 56). ¶Pooled serum samples from infants who received 3 doses of vaccine at 2, 4, and 6 months of age and had a blood sample taken 1 month after the last dose of vaccine (n = 47). #Pooled serum samples from infants who received 3 doses of vaccine at 2, 4, and 6 months of age plus a booster at 12 months and had a blood sample taken 1 month after the booster (n = 72). **Pooled serum samples from an additional cohort of infants who also received 3 doses of vaccine at 2, 4, and 6 months of age plus a booster at 12 months and had a blood sample taken 1 month after the booster (n = 94).

## Conclusions

The ability of the antibodies raised by Bexsero antigens to induce SBA activity against any given meningococcal isolate depends on the presence, level of surface expression, and sequence diversity of the respective antigens. Bexsero strain coverage can be predicted by using the Meningococcal Antigen Typing System, an ELISA which measures the level of antigen expression and antigenic diversity compared with the antigen in the vaccine (*9*). However, the correlation between the relative potency estimated by this typing system and the ability of a meningococcal isolate to be killed by serum from immunized persons has only been defined for MenB strains. 

We found that MenW:cc11 isolates causing invasive disease in England and Wales possessed alleles for NadA-2/3 peptide variants that are predicted to be highly cross-protective with the Bexsero NadA variant (*10*). The isolates also possessed alleles for NHBA peptide 29 which, although different from peptide 2 in Bexsero, has the potential to induce cross-protection even if NHBA-containing cross-protective epitopes have not been defined yet. Antibodies against Bexsero antigens can act synergistically and, therefore, the complement-mediated bactericidal killing observed could be mediated by antibodies against NadA, NHBA, or both.

In England, the ongoing MenW increase is similar to the MenC:cc11 outbreak in the mid-1990s, which was eventually controlled through mass vaccination (*11*). The increase in MenW cases has led to the rapid introduction of a national adolescent MenACWY conjugate vaccination program in August 2015 (https://www.gov.uk/government/news/new-meningococcal-vaccination-programme-expected-to-save-lives). In adolescents, a single MenACWY dose would provide direct protection and, by targeting the age group most likely to carry meningococci (*12*), could also provide indirect protection against MenW (Figure) and the other 3 capsular groups by reducing carriage and onward transmission to others.

**Figure:**
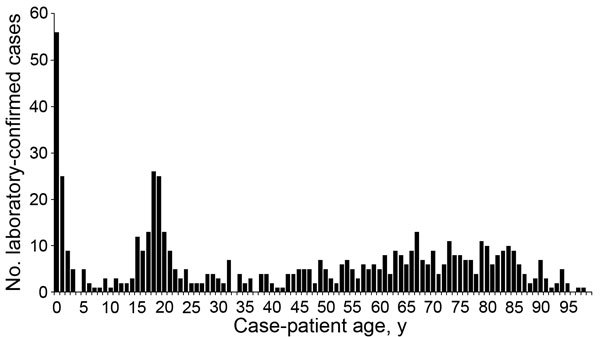
Age distribution of all laboratory-confirmed, invasive *Neisseria meningitidis* serogroup W disease cases identified in England during July 2009–December 2014.

Conversely, infants would require >2 MenACWY doses starting at 2 months of age, because MenW cases increase from birth and peak at 7 months of age before declining. Bexsero, which is predicted to protect against 73%–88% of invasive MenB isolates in England and Wales (*9*,*13*), could offer additional protection against MenB, which causes more cases among infants and toddlers than the other meningococcal serogroups. Among infants (<1 year of age), 101 MenB cases were reported during 2014–2015, compared with 21 MenW, 4 MenY, and 1 MenC; among toddlers, (1–4 years of age), 139 MenB cases were reported, compared with 18 MenW, 5 MenY, and 0 MenC. The difference in adolescents (15–19 years of age) is less pronounced, but 36 MenB cases were reported in this age group during 2014–2015, compared with 25 MenW, 14 MenY and 3 MenC cases (https://www.gov.uk/government/uploads/system/uploads/attachment_data/file/476989/hpr38-3915.pdf). However, the effectiveness of Bexsero against meningococcal carriage and, therefore, its ability to provide herd protection, which is a major objective of an adolescent programme, is less certain than with conjugate vaccines (*14*). These observations and our results support the recent implementation of both the adolescent MenACWY conjugate and infant MenB immunization programmes in the UK.
